# Last-Century Forest Dynamics in a Highland Pyrenean National Park and Implications for Conservation

**DOI:** 10.3390/plants13081144

**Published:** 2024-04-19

**Authors:** Valentí Rull, Arnau Blasco, Javier Sigro, Teresa Vegas-Vilarrúbia

**Affiliations:** 1 Botanic Institute of Barcelona, Spanish National Research Council (CSIC), Pg. del Midgia s/n, 08038 Barcelona, Spain; 2 Institut Català de Paleontologia Miquel Crusafont, Universitat Autònoma de Barcelona, C. de les Columnes s/n, ICTA-ICP Bld., 08193 Cerdanyola del Vallès, Spain; 3 Department of Evolutionary Biology, Ecology and Environmental Sciences, Universitat de Barcelona, Av. Diagonal 643, 08028 Barcelona, Spain; 4 C3 Centre for Climatic Change, University Rovira i Virgili, C. Joanot Martorell 15, 43480 Vilaseca, Spain

**Keywords:** national parks, paleoecology, forest dynamics, climate change, anthropogenic impact, conservation

## Abstract

Ecological records from before and after the creation of natural parks are valuable for informing conservation and management but are often unavailable. High-resolution paleoecological studies may bridge the gap and provide the required information. This paper presents a 20th-century subdecadal reconstruction of vegetation and landscape dynamics in a national park of the Pyrenean highlands. The park lands had traditionally been used for cultivation, extensive grazing, forest exploitation, and hydroelectricity generation following the damming of numerous glacial lakes. A significant finding is that forests have dominated the landscape, with negligible changes in composition, and only experienced fluctuations in forest cover, influenced by both climatic and anthropogenic factors. The creation of the park (1955) and the initial restrictions on forest exploitation did not significantly affect vegetation cover or composition. Major forest expansion did not occur until several decades later, 1980, when the park was enlarged and forest exploitation was further restricted. This expansion peaked in the 1990s, coinciding with a warming trend and a decrease in fire incidence, before declining due to warmer and drier climates. This decline was coeval with the ongoing global forest dieback and may be exacerbated by the predicted global warming in this century, which could also increase fire incidence due to dead-wood accumulation. Currently, the main threats are global warming/drying, fire, and tourism intensification. Similar high-resolution paleoecological records in protected areas are globally scarce and would be capable in providing the long-term ecological scope required to properly understand forest dynamics and optimize conservation measures.

## 1. Introduction

Understanding the dynamics of vegetation and landscape over the last few centuries, influenced by both climate change and human management, is crucial for optimizing conservation and restoration efforts. However, long-term ecological studies spanning 50 years or more are exceedingly rare, which highlights the value of high-resolution paleoecological records derived from recent sediments [1,2,3,4,5]. Natural parks and other areas designated for protection in the 20th century are particularly well-suited for these studies. Recent wetland sediments can provide detailed accounts of ecological changes following the introduction of conservation measures. These insights are useful for shaping management policies, offering precise tools for improved environmental stewardship at each site. Indeed, site-specific studies have proven to be highly beneficial, as they leverage paleoecological insights to inform conservation strategies by shedding light on ecosystem dynamics, ecological responses, and critical thresholds [4].

In the Iberian Pyrenees, there are two highland national parks—“Aigüestortes i Estany de Sant Maurici” (PNAESM) and “Ordesa y Monte Perdido” (PNOMP)—alongside other protected sites (natural parks), which boast numerous lakes and peat bogs containing records spanning from the Lateglacial to the present. However, high-resolution paleoecological records from the last century are scarce. The most detailed 20th-century Pyrenean record, featuring an average of approximately six years per sampling interval, was obtained from the annually laminated (varved) sediments of the mid-elevation Lake Montcortès (1027 m a.s.l.) [6]. Other records covering the last few centuries typically have resolutions at centennial or multicentennial time scales. Therefore, no high-resolution paleoecological records from the last century that may inform conservation are available for the PNAESM or the PNOMP.

This study focuses on the PNAESM, established in the mid-20th century. The specific target is Lake Sant Maurici, the park’s most iconic lake, from which a sediment core representing approximately the last century was obtained and analyzed palynologically at subdecadal resolution (averaging 3.5 years per sampling interval). The main objectives of this research were (1) to reconstruct the shifts in vegetation and landscape, with a particular emphasis on forest dynamics and fire incidence; (2) to correlate these trends with available climatic data and documented human activities, especially forest exploitation, grazing, and hydroelectricity generation; (3) to evaluate the impact of lake damming and the establishment of the park on the dynamics of vegetation and landscape; (4) to assess the resilience of highland forests against both natural and anthropogenic ecological pressures; and (5) to utilize the findings to inform conservation practices aimed at enhancing governance and management of the park. This high-resolution paleoecological reconstruction offers a unique record of vegetation dynamics and associated ecological drivers, unavailable through modern ecological studies. Additionally, this record falls within a temporal window that facilitates the integration of modern ecological and paleoecological data, thus establishing the way for the creation of continuous and truly long-term ecological datasets. Such records are more informative for conservation actions than short-term ecological studies.

## 2. Study Area

### 2.1. General Description

**Figure 1:**
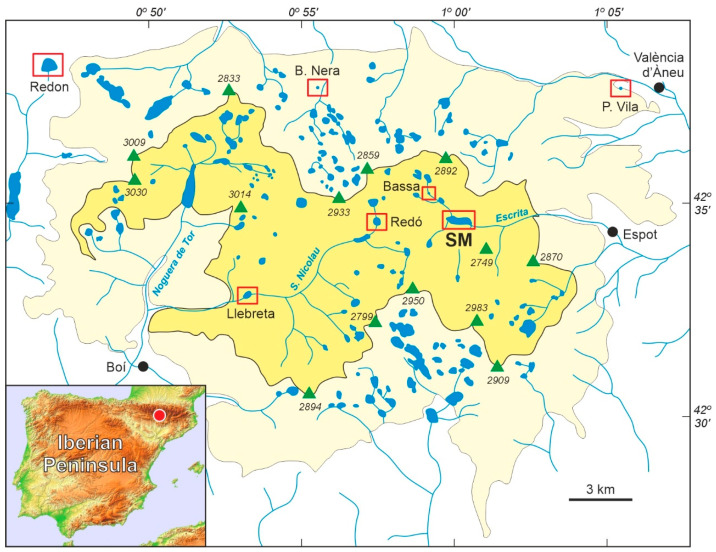
Sketch map of the Pyrenean “Aigüestortes i Estany de Sant Maurici” National Park (PNAESM) and its location in the Iberian Peninsula (red dot). The park itself is in yellow and the peripheral protection zone is in light yellow. Green triangles represent the highest peaks (elevations in m), and lakes and rivers are in blue. The localities (lakes, peat bogs) with Lateglacial/Holocene pollen records are highlighted by red boxes (SM, Lake Sant Maurici). Redrawn and modified from Ref. [10].

The PNAESM (Figure 1) was created in 1955, initially covering an area of 9851 ha. Before this, the lands were privately owned and the main activities were subsistence agriculture, extensive grazing, transhumant herding, hunting, fishing, and gathering. By 1975, forest exploitation had been restricted, but hydroelectricity generation—which began in 1910 and reached its peak from 1946 to 1960—and extensive grazing continued. In some sectors of the park, however, forest exploitation continued, due to previous agreements with private owners [7,8]. Hydroelectric power was generated by damming and underground drilling of a number of the abundant (>270) alpine lakes of glacial origin, situated at elevations between approximately 2000 and 2500 m [9]. However, the hydroelectric plants are located outside the park’s boundaries. Sant Maurici, one of the lakes impacted, was dammed in 1953, two years prior to the park establishment. Between 1985 and 1996, the park’s lands were reclassified as public and its area expanded to its current size of 14,119 ha, with an additional peripheral protection zone of 26,733 ha (Figure 1). Under this new administrative regime, the exploitation of natural resources, except for hydroelectricity and animal husbandry, became more restricted, particularly forest exploitation. Since the creation of the park, conflicts among the different actors involved—especially political/administrative sectors and private/communal owners—have been continuous due to the different interests and perceptions on how nature and natural resources should be managed [7,8].

Topographically, the PNAESM ranges from approximately 1600 m (1300 m including the peripheral buffer zone) to the highest peaks, which rise slightly above 3000 m [11]. Geologically, the park predominantly consists of Paleozoic formations, including Ordovician, Silurian, Devonian, and Carboniferous shales, interspersed with abundant Carboniferous granitic intrusions. The current landscape has been primarily sculpted by glacial activity during the Pleistocene. This glacial action has forged a distinctive glacial landscape characterized by features such as horns, ridges, cirques, U-shaped valleys, “roches moutonées”, moraines, and lakes, all superimposed on the Paleozoic bedrock [12].

According to the record from the AEMET-9660 meteorological station, located near the lake at an elevation of 1920 m, the average annual temperature is 5.4 °C, and the average annual precipitation is 1058 mm for the period 1988–2017. The highest average monthly temperatures, slightly above 14 °C, occur in summer (July–August), while the lowest, approximately −1.5 °C, are observed in winter (January–February). In terms of precipitation, monthly averages consistently exceed 60 mm, peaking in May (188 mm) and November (110 mm). The lowest precipitation levels occur in February (63 mm) and July (65 mm) [10]. Additionally, an integrated instrumental climatic record for the entire PNAESM during the 20th century has been compiled recently [13], covering a substantial portion of the timeframe addressed in this study.

The general distribution of vegetation is primarily governed by marked elevational gradients in climatic parameters, especially temperature, which decreases at an average rate of 0.6 °C per 100 m in elevation [11]. The montane belt, ranging from 1200 to 1600/1800 m and predominantly found in the peripheral zone, features deciduous and mixed forests with species such as *Abies alba* (silver fir), *Fagus sylvatica* (European beech), *Corylus avellana* (common hazel), *Betula pendula* (silver birch), *Fraxinus excelsior* (common ash), and *Pinus sylvestris* (Scots pine). The subalpine belt, spanning 1600 to 2300/2500 m, mainly consists of conifer forests with *Abies alba* and *Pinus mugo* subsp. *uncinata* (mountain pine, also known as *P. uncinata*), with the upper boundary of this belt marking the treeline, which is located between 2300 and 2500 m elevation, varying with local slope characteristics. Above this treeline lies the alpine belt, dominated by *Festuca eskia* grasslands. The alpine wetlands, encircling lakes and bogs, are dominated by species such as *Carex nigra*, *Eriophorum angustifolium*, and *Scirpus cespitosus*. Beyond 2700 m, in the subnival zone, cushion-like plants, including *Saxifraga* spp. and *Silene acaulis*, can be found thriving in rocky areas [11].

**Figure 2:**
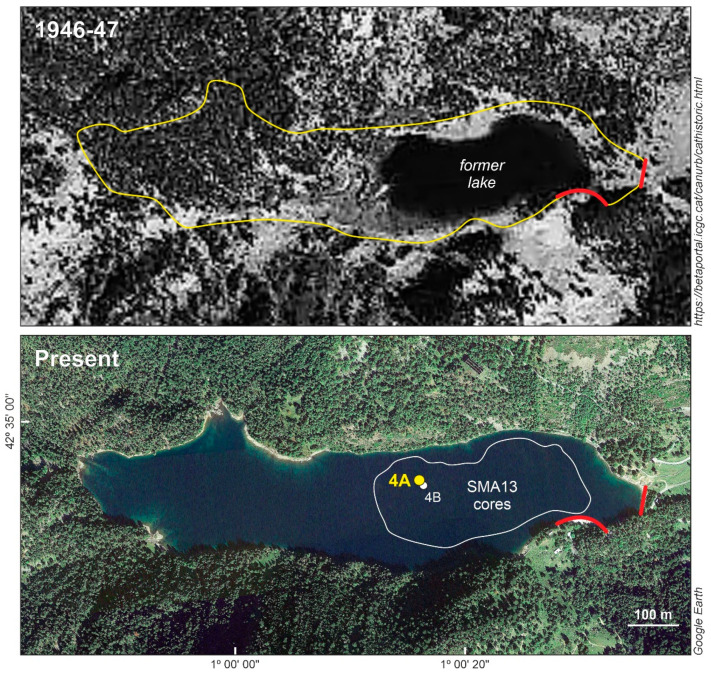
Lake Sant Maurici before and after damming (1953 CE). The upper panel shows the former glacial lake and the contour of the present reservoir (yellow line), whereas the lower panel displays the present reservoir and the contour of the former lake (white line). Damming areas are indicated by red lines. The core used in this study (SMA13-4A) is highlighted in yellow and the core studied by Calero et al. [15] and Rull et al. [10] (SMA13-4B) is indicated in white. See Ref. [16] for more details.

Lake Sant Maurici (42°34′53″ N, 1°00′12″ E; 1914 m elevation) (Figure 1) is located in a glacial cirque within the subalpine belt, surrounded by the typical high-altitude conifer forests of mountain pine and silver fir. Originally, the lake was much smaller and shallower than it is today. In 1953 CE, the construction of a 16 m high dam transformed this small pond into a reservoir approximately 1 km long and 200 m wide (Figure 2), with a maximum depth of 25 m. This reservoir is now used for hydroelectricity generation, irrigation, and human consumption [9]. The damming and related construction led to the removal of vegetation around the dam and the introduction of ruderal species, increased soil erosion around the access routes, and flooded the surrounding terrains. This flooding irreversibly destroyed the basin floor’s vegetation not adapted to inundation and water-level fluctuations, primarily affecting conifer forests and peat bogs along the lake margins, which were habitats for many unique floristic elements of the Pyrenean highlands [14]. The aquatic ecosystems also suffered significantly from the raised water levels, eutrophication, and oxygen depletion due to the decomposition of dead plant biomass [9].

### 2.2. Previous Studies

Palynological studies conducted in the PNAESM and its surroundings (Figure 1) have reconstructed the Lateglacial and Holocene vegetation dynamics with multidecadal to centennial resolution [17,18,19]. In Sant Maurici, a 9 m deep core (SMA13-4B) was retrieved from the edge of the former glacial lake (Figure 2). This core contains a fragmentary Holocene record, due to sedimentation interruptions caused by water-level fluctuations [15]. The Late Holocene section of this core encompasses the Bronze Age (4.3–3.5 cal kyr BP) and the Middle Ages (1.5–0.3 cal kyr BP), with a significant sedimentary gap omitting the interval corresponding to the Iron Age and the Roman period (3.5–1.5 cal kyr BP). The analysis revealed that the highland forests around the lake maintained stability in abundance and composition, even through temperature and moisture shifts associated with the Bronze Age Warm Period (BAWP), the Dark Ages Cold Period (DACP), the Medieval Climate Anomaly (MCA), and the Little Ice Age (LIA). Human activities in the area were intermittent and of low intensity, exerting minimal impact on the forest dynamics [10].

Therefore, unlike most highland Pyrenean landscapes, which experienced extensive deforestation and anthropogenic transformation from the Bronze Age to the Middle Ages, the forests around Sant Maurici demonstrated remarkable constancy over time. It has been suggested that Sant Maurici may have served as a microrefugium for highland forests during the Late Holocene [20]. A 1 m deep gravity core, retrieved from the same location (SMA13-4B-1G), provided a record spanning the last 0.7 cal kyr BP. This record includes a significant gap between 0.6 cal kyr BP and the present, but offers a complete record of the last century in the upper 22 cm. However, high-resolution pollen analysis of this segment was hindered by the scarcity of material remaining after sampling for ^210^Pb dating; only four samples were analyzed, which proved insufficient for a detailed reconstruction of vegetation and landscape dynamics during the 20th century.

## 3. Methods

### 3.1. Coring and Dating

The Sant Maurici core used in this study (SMA13-4A; 141 cm depth) was collected in October 2013 using a UWITEC gravity corer with a diameter of 63 cm, at a water depth of approximately 18 m, in the vicinity of the former glacial lake, near the previously mentioned parallel core SMA13-4B (Figure 2). The dating was conducted at the Department of Physics of the Autonomous University of Barcelona. ^210^Pb was analyzed under the assumption of secular equilibrium with its daughter ^210^Po. Following microwave-assisted acid digestion, ^210^Po was quantified using alpha spectrometry [21]. To calculate the sedimentation rate, the constant rate of supply (CRS) model for ^210^Pb was applied [22]. Supported ^210^Pb levels were determined from a composite of three deep samples using gamma spectrometry, specifically through the measurement of ^214^Pb emission lines at 295 keV and 352 keV, assuming secular equilibrium with ^226^Ra.

### 3.2. Pollen Analysis

For pollen analysis, 25 samples of 2 cm^3^ were collected at regular intervals using a volumetric syringe. These samples underwent digestion with NaOH for disaggregation, HCl for carbonate removal, and HF for silicate removal, followed by centrifugation with Thoulet solution (density 2 g·cm^−3^) for mineral separation [23]. Prior to processing, two tablets of *Lycopodium* spores (Batch 280521 291; 13,761 ± 448 spores/tablet) were added to each sample [24]. The processing took place at the Archaeobotany Lab of the Catalan Institute of Human Paleoecology and Social Evolution (IPHES). Pollen and spore identification was based on Refs. [25,26] and the author’s (VR) own reference collection, which comprises over 700 species/subspecies from the Iberian flora. Nonpollen palynomorph (NPP) identification followed López-Vila et al. [27] and the NPP Image Database (accessible at https://non-pollen-palynomorphs.uni-goettingen.de/, last visited 3 May 2023). While fungal spores were generally not identified, those from coprophilous fungi, indicating grazing and hence human impact, received special attention [28,29,30,31]. Spores of *Glomus*, a mycorrhizal fungus indicative of soil erosion following local forest clearance in the study area, were also identified [27]. Charcoal particles served as proxies for the fire regime and were categorized into two main size groups to differentiate between regional fires (<150 µm) and fires occurring near the coring site (>150 µm) [32].

Counting was conducted until reaching a minimum pollen sum of 300 grains, along with the stabilization of confidence intervals and saturation of diversity [33]. In several samples, *Pinus* pollen was overwhelmingly abundant, with counts halted at 100–150 grains. Beyond this point, counts were extrapolated using *Lycopodium* spores. For these samples, the specified statistical criteria were applied to the non-*Pinus* pollen types. The pollen sum excluded aquatic and semiaquatic plants (Cyperaceae, *Epilobium/Oenothera*, *Ranunculus*) to focus on terrestrial vegetation. Consistent with standard practices in pollen analysis, percentages were calculated to deduce vegetation composition, while pollen accumulation rates (PAR) served as proxies for plant cover [34].

## 4. Results and Interpretation

### 4.1. Chronology and Sedimentation

**Figure 3:**
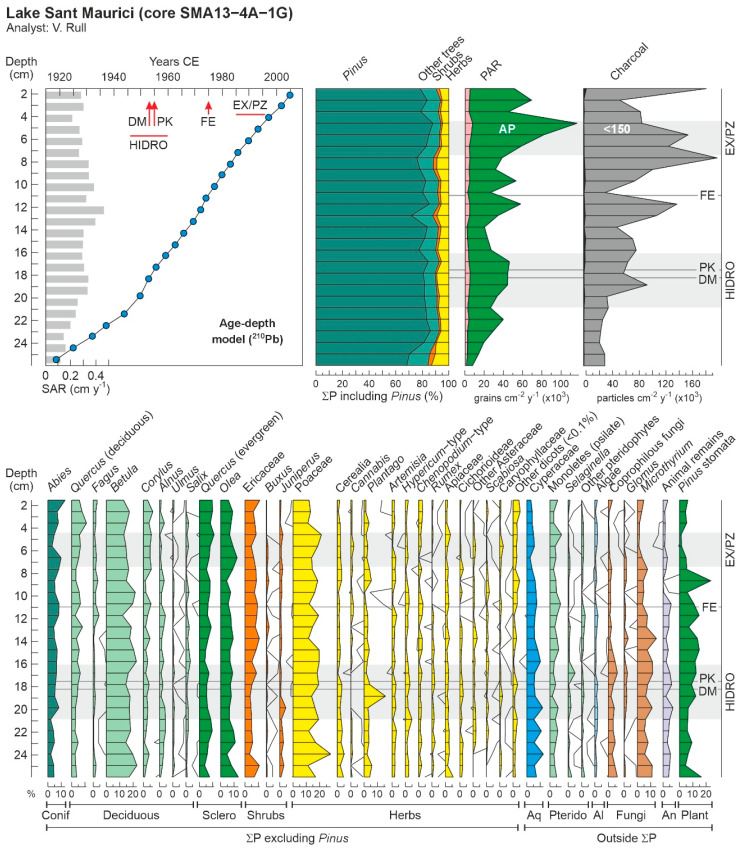
Age–depth model and pollen diagrams for the Sant Maurici core SMA13-4A-1G. The upper panel shows the age–depth model (Table 1), the summary percentage diagram using the main pollen sum, which includes *Pinus*, the pollen accumulation rates (PAR) of arboreal (green) and nonarboreal pollen (pink), and the charcoal influx (particles larger than 150 μm in black and the others in gray). Abbreviations: HIDRO, maximum development of hydroelectrical exploitation; DM, damming of Lake Sant Maurici; PK, creation of the PNAESM; FE, restriction of forest exploitation; EX/PZ, park expansion and creation of the protection zone. The lower panel displays the abundance of palynomorphs other than *Pinus*, with respect to the reduced pollen sum (excluding *Pinus*), using the pollen types above 0.1% of the total. Other dicots (<0.1%) include *Castanea*, *Juglans*, *Tilia*, *Acer*, *Ilex*, *Ephedra*, *Helianthemum*, *Galium*, *Centaurea*, *Ribes*, *Valerianella*, *Geranium*, *Malvaceae*, *Lupinus*, *Phyteuma*, *Filipendula*, *Urtica*, *Drosera*, *Epilobium/Oenothera*, and *Ranunculus*. Other pteridophytes include *Asplenium*, *Thelypteris*, *Isoetes*, *Polypodium*, *Sphagnum*, *Cryptogramma*, and psilate triletes. Algae include *Pediastrum*, *Spirogyra*, *Staurastrum*, *Bulbochaete*, *Botryococcus*, *Mougeotia*, and *Debarya*. Coprophilous fungi are *Sporormiella*, *Sordaria*, and *Podospora*. Animal remains include chironomids, tardigrada (*Macrobiotus*), rotifers (*Habrotrocha*), acari, cladocera, tecamoebae (*Arcella*), and platelminthes (*Neorhabdocoela*) (Figure 4).

The results of the ^210^Pb dating are presented in Table 1. Samples with dating errors exceeding the sampling intervals (more than ±6 years) were excluded from this study. Consequently, the period analyzed spans 88 years, from 1919 to 2007 CE, with an average resolution of 3.5 years. While sample 20P was utilized for pollen analysis, it was not used for dating purposes and its age was interpolated. The age–depth model is illustrated alongside the pollen diagram in Figure 3 to enhance visualization. Sediment accumulation rates averaged 0.285 cm per year (cm/y), displaying an increasing trend that peaked at 0.463 cm/y around the mid-section (11.2 cm depth, corresponding to 1974 CE) before declining. To aid in comparison, the dates of significant human activities discussed in Section 2.1 are marked on the chronological scale derived from the age–depth model, as shown in Figure 3.

### 4.2. Vegetation Dynamics

**Figure 4:**
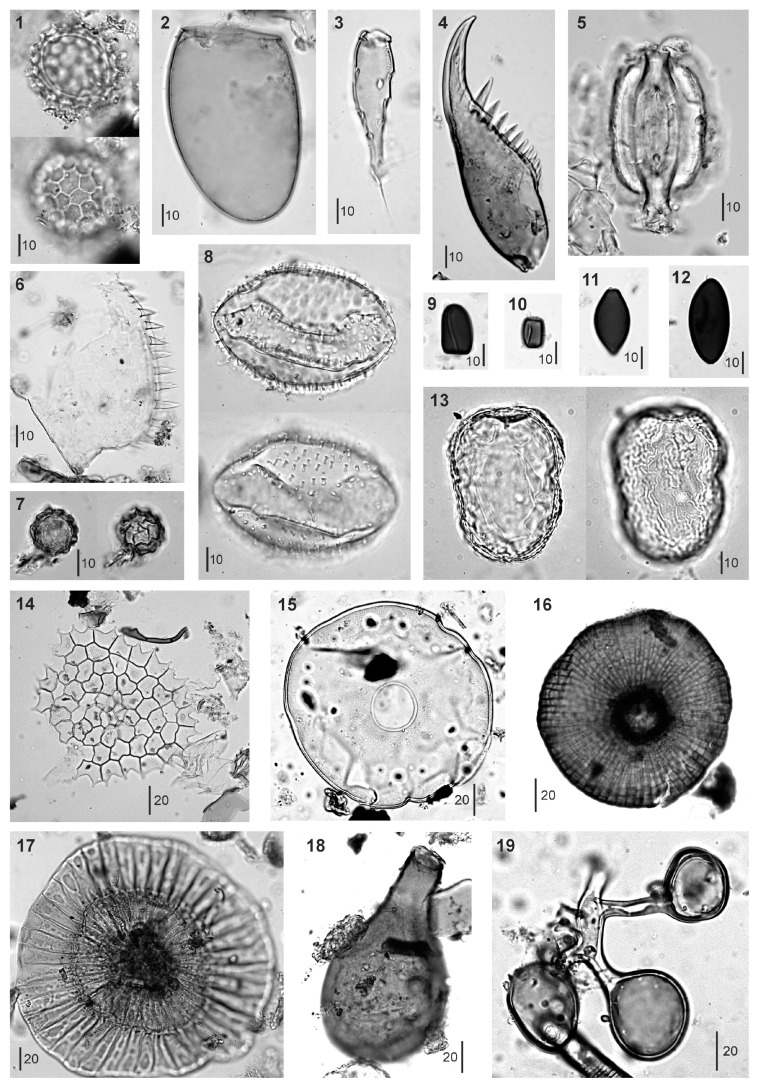
The main NPP (nonpollen palynomorphs) identified in Sant Maurici core SMA13-4A-1G. 1, NPP16; 2, *Neorhabdocoella* egg (tardigrada); 3, Acari leg; 4, Chironomid mandible; 5, *Pinus* stoma; 6, Cladocera mandible; 7, NPP7; 8, NPP14; 9–10, *Sporormiella* spores (fungi); 11, *Sordaria* spore (fungi); 12, *Podospora* spore (fungi); 13, NPP18; 14, *Pediastrum* coenobium (algae) 15, *Arcella* shell (tecamoebae) 16–17, *Microthyrium* ascomata (fungi); 18, *Habrotrocha* shell (rotifera); 19, *Glomus* spores (fungi). Bar sizes in μm.

In total, 90 palynomorph types were identified and counted: 56 types were pollen, 9 were pteridophyte spores, and 25 were NPP (8 algae, 5 fungi, 7 animals, 1 plant, and 4 of unknown origin). The most abundant NPP types are depicted in Figure 4 to support future research in the area, where studies on NPP are scarce. The stratigraphic distribution of these palynomorphs is illustrated in the integrated pollen diagram (Figure 3). *Pinus* pollen was overwhelmingly dominant, showing minimal variation (accounting for 81 ± 10% of the main pollen sum, which averaged 1274 grains). To mitigate potential masking effects on the visibility of other pollen types, a reduced pollen sum was calculated by excluding *Pinus*, resulting in an average of 233 grains.

A review of the pollen diagram, whether considering the main or the reduced pollen sum, quickly reveals a remarkable constancy in vegetation composition over time (Figure 3). Indeed, conifer forests, predominantly composed of *Pinus*, have extensively covered the catchment throughout the 20th century. Similarly stable were the abundances of *Abies*, the second most significant tree species in these forests, and *Rhododendron*, the predominant understory shrub, represented in the diagram by Ericaceae pollen. This constancy also extends to trees from lower elevations, primarily deciduous species from the montane forest belt (notably *Betula*, *Corylus*, and deciduous *Quercus*), as well as lowland species from Mediterranean sclerophyll forests, such as evergreen *Quercus* and *Olea*. The primary observable changes were in plant cover, as suggested by pollen accumulation rates (PAR), and fire regime, as indicated by charcoal influx. PAR values, largely influenced by arboreal pollen, serve as a reliable proxy for total forest cover. Notably, almost all charcoal particles were smaller than 150 µm, making them effective indicators of regional fire trends.

**Figure 5:**
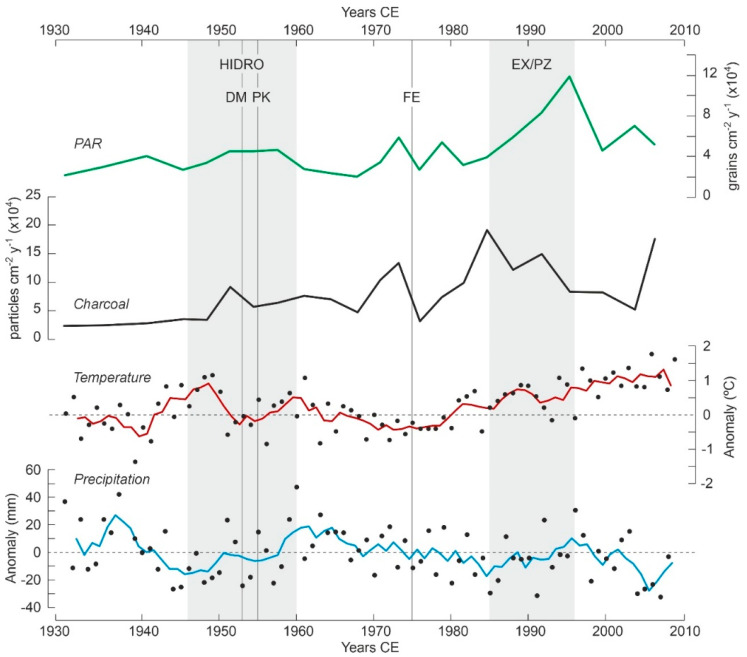
Comparison of pollen accumulation rates (PAR) as a proxy for forest cover, and charcoal influx, as a proxy for fire incidence, with annual average temperature and precipitation trends for the PNAESM. Climatic values are expressed as anomalies and their trends are smoothed using a 4-year moving average (raw data from Ref. [13]). Abbreviations as in Figure 3: HIDRO, maximum development of hydroelectrical exploitation; DM, damming of Lake Sant Maurici; PK, creation of the PNAESM; FE, restriction of forest exploitation; EX/PZ, park expansion and creation of the protection zone.

The diagram starts in the early 1910s, coinciding with the onset of hydroelectricity exploitation within the PNAESM, a period marked by a minor increase in forest abundance and cover, which stabilized around 1940, just before the hydroelectric industry reached its peak development. This rise in conifer forests was accompanied by a slight decline in other forest elements, notably *Betula*, known for successfully colonizing forest clearings in the subalpine belt [35]. It is conceivable that the forest recovery, following an initial clearing not captured in the diagram, was facilitated by a reduction in fire incidence, which remained at minimal levels during this time. During this phase, temperatures hovered around the average for the 1930–2020 period, while precipitation experienced a short increase, peaking in the late 1930s (Figure 5). This climatic condition likely supported the observed forest increase. Thus, the modest forest growth noted up until the 1940s may have been the result of a synergistic effect of increasing precipitation and reduced fire incidence. After 1940, forest cover reverted to previous levels, coinciding with a rise in temperatures and a decrease in precipitation.

During the peak period of hydroelectric exploitation (1946–1960), the vegetation composition remained stable, and forests saw a second small recovery, this time solely in terms of plant cover. A notable increase in *Plantago* around the time of the lake damming (1953) likely reflects the transient surge in ruderal vegetation due to local disturbances from the dam construction activities. The establishment of the PNAESM in 1955 did not result in discernible shifts in vegetation composition. Interestingly, an increase in fire incidence coincided with the growth in forest cover, which might be attributed to an increase in available plant fuel for combustion. However, this hypothesis requires further evidence for conclusive validation. These periods of increased plant cover and fire incidence occurred alongside trends of decreasing temperatures and average precipitation, suggesting a minimal climatic influence on these patterns.

During the 1960s, there was a reduction in plant cover without significant changes in the composition of either forested or nonforested vegetation. This period was characterized by low to intermediate fire incidence and a slight decrease in temperature and precipitation, suggesting that the interplay between climate and fire might have played a significant role. Notably, the period also saw a reduction in coprophilous fungi, particularly *Sporormiella*, indicating a decline in grazing activities. This reduction could reflect a broader decrease in grazing within the PNAESM or result from the local flooding of pastures following dam construction. A third resurgence in forest cover was observed shortly before the imposition of restrictions on forest exploitation in 1975, with no notable changes in community composition. This increase in forest cover once again occurred alongside a significant peak in fire incidence, lending support to the potential link between fire intensity and fuel availability discussed earlier. The lack of significant shifts in temperature and precipitation, which remained stable around the average, bolsters this interpretation.

The imposition of general restrictions on forest exploitation, in 1975, was paralleled by an initial decline in forest cover, followed by an increase and then another decline by 1980. This initial decline occurred alongside a significant reduction in fire incidence, which then surged, reaching its highest levels in the mid-1980s. The fluctuating forest cover values indicate that the forests did not immediately expand in response to the cessation of forest exploitation. It is also possible that the strong fire increase would have contributed to reducing forest cover thus compensating for an eventual forest expansion due to the exploitation cessation. We will come back to this point later. Climate likely played a secondary role in these dynamics, as temperatures were slightly rising and precipitation levels were relatively stable, despite a notable drop coinciding with the peak in fire incidence in the mid-1980s.

During the period of park expansion and the establishment of the peripheral protection zone (1985–1996), forest cover experienced a major increase, reaching its peak in the mid-1990s. The composition of the forest remained constant throughout this time. This increase in forest cover coincided with a notable decline in fire incidence, alongside rising temperatures and increasing precipitation. This pattern suggests that the reduction in fire incidence, combined with climatic conditions conducive to forest growth, supported the expansion of forest cover. At the start of this phase, a simultaneous decline was observed in *Pinus* stomata and *Microthyrium* ascomata. These NPP exhibited very similar trends throughout the entire diagram, indicating a potential relationship between them. *Microthyrium*, a genus of saprophytic fungi that thrives on leaf litter [36], and *Pinus* stomata, which signal the presence of leaf tissue fragments of local origin in sediments [37], can both serve as indicators of sedimentary leaf litter accumulation, particularly from *Pinus*.

Determining the precise causes behind the inferred decline in leaf litter observed in Sant Maurici sediments during the 1980s, and its chronological alignment with the expansion of the park and the creation of the peripheral protection zone, remains challenging given the current evidence. Notably, the surge in *Pinus* stomata immediately following its sharp decline aligns with an uptick in *Plantago* pollen from 1980 onwards. *Plantago* has been identified as an indicator of local disturbance, notably during dam construction activities mentioned earlier. Around this time, park infrastructures underwent renovation and enhancement, with improvements made to access routes, likely contributing to local vegetation disturbances. Consequently, there was a significant rise in visitor numbers, which could account for the sustained increase in *Plantago* presence up to the present. However, there appears to be no direct link between these developments and the significant decline in leaf litter during the 1980s.

A potential explanation for the observed phenomena might relate to the regime of forest exploitation. Logging, particularly for wood extraction, significantly increases the volume of leaf litter on the forest floor by leaving cut branches behind. A portion of this litter eventually makes its way into the lake and its sediments, thereby increasing the content of cuticles where stomata and *Microthyrium* ascomata remain after the decay of more labile organic matter. Consequently, these residues could serve as indicators of forest exploitation. Their continued presence beyond 1975, the year when such practices were officially restricted, suggests that actual cessation of forest exploitation might not have occurred until 1980, coinciding with the implementation of stricter restrictions. This timeline aligns more closely with the amazing increase in forest cover—possibly resulting from either effective expansion of the forested area, an increase in tree density, or both—and the notable decrease in fire incidents starting from 1980.

Following park expansion, there was another decline in forest cover, which occurred alongside a period of low fire incidence (with the exception of the last sample, indicating a recent fire increase), rising temperatures, and decreasing precipitation. It is plausible that, with the increased restrictions on human activities, climatic factors became the dominant force. The warming and drying trend observed since the mid-1990s may have contributed to the reduction in forest cover, which attained values similar to the 1970s.

## 5. Discussion and Conclusions

### 5.1. Summary of Main Trends

The above results indicate that the composition of both forested and nonforested vegetation within the Lake Sant Maurici catchment area remained relatively stable throughout the 20th century, demonstrating significant resilience to changes in climate and human activities. This resilience mirrors patterns observed during the Bronze Age and Middle Ages when the catchment area was proposed to have served as a microrefugium for forests amidst widespread deforestation for grazing expansion in the Pyrenean highlands [10,20]. However, human impacts during these earlier periods were limited to low-intensity activities such as forest clearing for local cultivation and grazing by nomadic or seasonally migrating societies. In contrast, the 20th century saw a marked intensification of human activities within the PNAESM, including widespread hydroelectric exploitation through lake damming, extensive grazing, forest management for wood extraction, and tourism development, among others. This intensification, coupled with shifts in temperature and precipitation, created a complex scenario of evolving natural and anthropogenic forces and their interactions. Despite these highly dynamic environmental conditions, the vegetation of Sant Maurici still exhibited remarkable resilience in terms of its composition. Climatic and human influences primarily affected plant cover, which was overwhelmingly dominated (90% or more of pollen content) by conifer forests resembling those of the present day, while nonforested communities played a minor role.

The lowest forest cover values were observed from the beginning of hydroelectric exploitation in the 1910s until the late 1970s. During this period, significant events such as the peak of hydroelectric industry activity, the damming of the lake, or the establishment of the national park appeared to exert little or no influence on forest dynamics. Instead, regional fires and climatic trends, along with their potential interactions, played a more pivotal role in the subtle fluctuations of forest cover. It was not until 1980, coinciding with a decline in fire incidence and the expansion of the park alongside stricter protection measures, that forest cover began to significantly increase. Warming trends during this period likely also facilitated forest expansion. In essence, anthropogenic factors emerged as the primary drivers of forest change, with climatic conditions acting as a modulating influence. Interestingly, human interventions such as the lake damming, the establishment of the PNAESM, and the initial restrictions on forest exploitation before 1975 did not reach a threshold of significant impact on forest cover. However, the park’s expansion and the intensification of exploitation restrictions from 1980 onwards surpassed this tipping point. In recent decades, the combination of warming and drying trends has played a crucial role in reducing forest cover, particularly in the absence of logging activities.

### 5.2. Comparisons with Other Localities and Proxies

Given the absence of comparable highland palynological records from the last century in the Pyrenees and across the Iberian Peninsula [38], the findings of this study can be compared with the mid-elevation Montcortès record and various dendrochronological analyses conducted within the PNAESM.

Lake Montcortès, located 28 km to the south within the lower montane belt, presents a stark contrast in both vegetation and human historical impact compared to Lake Sant Maurici. The regional vegetation around Lake Montcortès is a diverse mosaic comprising deciduous, mixed, and evergreen forests dominated by various oak and pine species, alongside meadows, pastures, and cultivated fields [39]. This region has experienced significant human disturbances since Roman times, marked by three major deforestation events that substantially reduced forest cover [6]. The present-day forests emerged through natural recovery following a 40% reduction in 1800 CE. Throughout the 20th century, these forests showed minimal compositional change, with the only notable shift being a general increase in forest cover, peaking shortly after 1980. Thus, the constancy in composition and variations in forest cover are shared characteristics between the Sant Maurici and Montcortès records. Further studies are required to determine if this pattern represents a broader regional phenomenon and to pinpoint the underlying drivers. A unique difference between the two records is the increase in *Cannabis* pollen at Montcortès during the 1980s, attributed to the extensive cultivation of hemp in the adjacent southeast lowlands [40], a feature not observed in the Sant Maurici record. This particular aspect warrants further investigation and will be explored in greater detail in the future through pollen dispersion modeling.

Dendrochronological studies in the PNAESM and its surrounding areas have primarily focused on reconstructing past climates [41], documenting snow avalanches [42], and analyzing tree-growth responses, particularly in *Pinus uncinata*, to environmental drivers, with a notable emphasis on climatic conditions. This discussion concentrates on the latter category of studies, especially those that can enhance our understanding of the paleoecological reconstruction of Sant Maurici. Over time, the growth of *P. uncinata* trees has been positively correlated with temperatures during the growing season, which includes spring and summer [43]. This correlation may help clarify some of the interpretations related to temperature increases and forest cover expansion observed in the study. However, responses to climatic conditions can vary among pine populations. For instance, mountain pine trees at lower elevations within the park are more adversely affected by summer drought conditions than those at higher elevations [44]. Given that Sant Maurici is situated at a higher elevation, the impact of summer drought on its forests is expected to be less significant compared to lower elevation areas. Consequently, temperature emerges as the primary factor influencing forest growth in this context, which supports previous interpretations.

Other factors, such as the duration of the winter snow season and the depth of the spring snowpack, also play a significant role in affecting pine growth. Indeed, longer snow seasons and deeper snowpacks have been shown to negatively impact tree growth, irrespective of the atmospheric temperatures during the growing seasons [45]. Consequently, warmer conditions, which lead to shorter winters and shallower spring snowpacks, could potentially benefit the growth of mountain pine trees. Once more, temperature emerges as a fundamental driver for forest growth.

**Figure 6:**
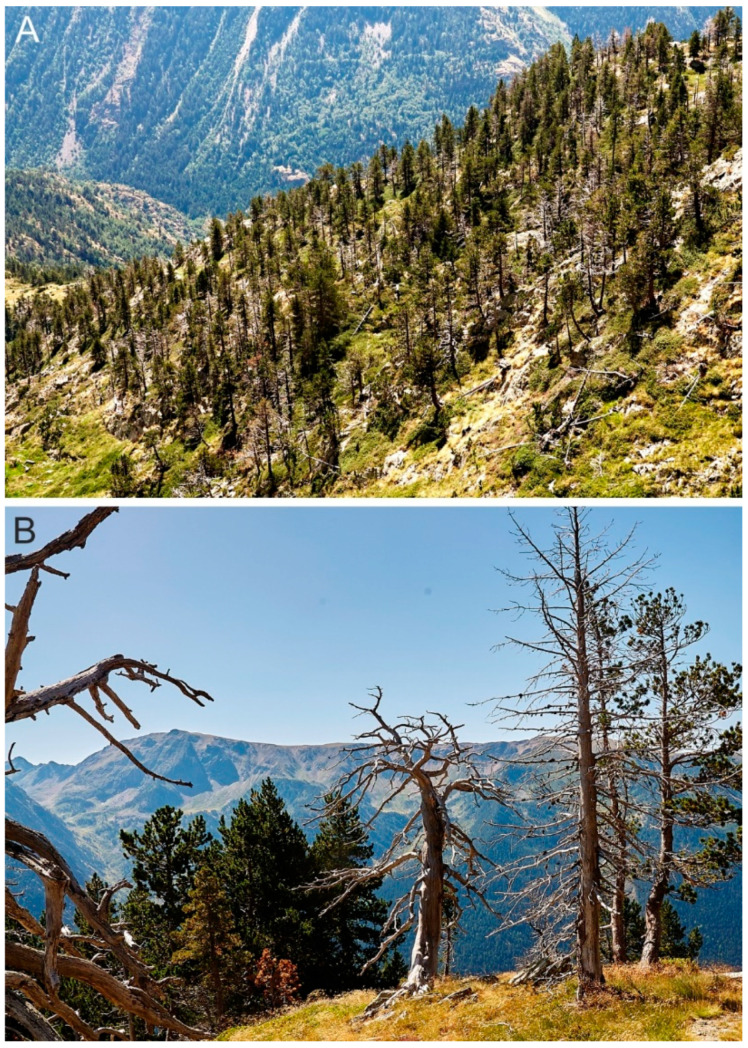
Mountain pine (*P. mugo* subsp. *uncinata*) dieback in the surroundings of Lake Naorte, situated 27 km NE from Lake Sant Maurici. (**A**) Mountain pine forest with a large proportion of dead trees. (**B**) Close-up of the same forest patch showing some dead pines and others alive with signs of drying (brown tones). Photos: V. Rull.

Recent dendroecological surveys have also demonstrated an increase in forest dieback on the Pyrenees linked to current warming and drought intensification [46,47]. This is a worldwide phenomenon that has intensified in the last few decades [48] and is clearly visible in the PNAESM and its surroundings (Figure 6). This massive forest dieback could help explain the reduction in forest cover observed in the Sant Maurici record starting at the mid-1990s and linked to warming and drying trends.

### 5.3. Conservation Insights

The high compositional resilience of the forests in Sant Maurici, coupled with their sensitivity to changes in forest cover due to environmental shifts, may have significant implications for conservation. The continuation of the warming and drying trends predicted by the IPCC for this century [49] is likely to exacerbate the reduction in forest cover. Based on the results presented, substantial compositional changes are not anticipated unless temperature and/or precipitation reach physiological thresholds critical to tree species with varying climatic tolerances.

A recent ecophysiological study in the PNAESM indicated that *Pinus uncinata* exhibits high tolerance to variations in precipitation, while *Betula pendula* is particularly vulnerable to low precipitation levels, and *Rhododendron ferrugineum* is adversely affected by brief drought events [50]. Consequently, continued drying and a potential increase in drought frequency could further solidify the dominance of mountain pine. Furthermore, if the anticipated reduction in forest cover leads to a decrease in tree density (Figure 6), *R. ferrugineum*, which thrives in the shade beneath mountain pine canopies, might be replaced by shrubs more resistant to sunlight exposure. Additionally, *B. pendula*, frequently found colonizing high-mountain forest clearings, might see an expansion in its populations as long as its moisture requirements are met. This suggests that conservation strategies should prioritize understanding the species-specific responses to potential warming and drying trends, rather than focusing solely on the forest as a whole.

A topic of considerable debate is whether ecosystems should be preserved in their current state or restored to a condition resembling their pre-anthropic state, as inferred by paleoecological records [3]. For Sant Maurici, the earliest paleoecological records available date back to the Bronze Age (ca. 4.3 to 3.5 cal kyr BP). These records show that the forest composition was remarkably similar to today and that human impact at that time was minimal [10]. This similarity indicates that the contemporary forest state closely mirrors its natural condition, suggesting that restoration efforts might not be as critical here as in other Pyrenean highlands. Historically, many of these areas were forested but have since been transformed into extensive pastures due to human activities [51].

Special attention must be given to fire incidence. The forest dieback observed over recent decades not only diminishes forest cover and impacts shadow-loving understory shrubs but also leads to a significant increase in highly flammable deadwood (Figure 6). The sharp rise in fire incidence seen in the early 2000s could stem from the accumulation of dead trees. Should the current trend of warming and drying persist, fires could pose a major threat to the forests within the PNAESM.

The findings of this study highlight that the most effective conservation strategies developed to date included establishing a peripheral buffer zone and implementing a strict ban on forest exploitation. Consequently, further expansion of the park, adhering to these same regulations, would likely be advantageous for conservation efforts. If such expansion encompasses areas that were once forested but have since been converted into pastures, restoration initiatives should be seriously considered. For these areas, conducting new paleoecological studies tailored to each specific catchment area would be essential. As it occurred with the creation of the PNAESM and the progress of conservation regulations, an eventual expansion of the park would enhance conflicts with private and communal owners [7,8]. Therefore, further negotiations among all the actors involved would be necessary.

This study demonstrates the utility of high-resolution paleoecological records to properly understand forest dynamics and the involved drivers (climate, fire, human management), in protected and non-protected areas where long-term ecological studies of the last centuries are scarce or unavailable. The lack of such ecological studies is common and detailed paleoecological reconstructions may be of much help to inform conservation and management practices.

## Figures and Tables

**Table 1:** Results of ^210^Pb dating using the CRS model and the depth of sample tops. SAR, sediment accumulation rates.

Sample	Depth (cm)	Excess ^210^Pb (Bq·kg^−1^)	Date (year)	SAR (cm·y^−1^)
1P	1.0	292.5 ± 11.1	2007 ± 1	0.282 ± 0.013
2P	2.0		2005 ± 1	0.301 ± 0.017
3P	3.1	360.4 ± 21.5	2002 ± 1	0.212 ± 0.015
4P	4.1		1997 ± 1	0.269 ± 0.017
5P	5.1	228.4 ± 11.1	1993 ± 1	0.291 ± 0.017
6P	6.1		1990 ± 1	0.266 ± 0.021
7P	7.1	164.9 ± 16.0	1986 ± 1	0.343 ± 0.037
8P	8.1		1983 ± 1	0.344 ± 0.040
9P	9.2	98.3 ± 11.5	1980 ± 1	0.384 ± 0.049
10P	10.2		1977 ± 1	0.322 ± 0.043
11P	11.2	76.4 ± 9.4	1974 ± 1	0.463 ± 0.062
12P	12.2		1972 ± 2	0.394 ± 0.051
13P	13.2	85.8 ± 9.2	1969 ± 2	0.303 ± 0.037
14P	14.3		1966 ± 2	0.298 ± 0.033
15P	15.3	91.1 ± 6.9	1963 ± 2	0.291 ± 0.029
16P	16.3		1959 ± 2	0.307 ± 0.038
17P	17.3	77.3 ± 9.9	1956 ± 2	0.341 ± 0.052
18P	18.3		1953 ± 2	0.333 ± 0.052
19P	19.9	52.5 ± 6.9	1950 ± 3	0.256 ± 0.043
20P ^1^	20.4		1947 ± 3	0.248 ± 0.042
21P	21.4	60.5 ± 7.6	1944 ± 3	0.239 ± 0.041
22P	22.4		1937 ± 4	0.199 ± 0.037
23P	23.4	61.8 ± 8.2	1932 ± 4	0.146 ± 0.030
24P	24.4		1925 ± 5	0.161 ± 0.038
25P	25.5	43.5 ± 7.7	1919 ± 6	0.120 ± 0.035
1C ^2^	27.0		1911 ± 8	0.070 ± 0.039
2C ^2^	28.0	34.8 ± 17.7	1889 ± 11	0.043 ± 0.037
3C ^2^	29.0		1865 ± 15	0.031 ± 0.031
4C ^2^	30.0		1832 ± 32	ND
5C ^2^	31.1	<0	ND	ND

^1^ Only for pollen analysis (interpolated age). ^2^ Not used in this work.

## Data Availability

Raw data are available on request to the first and last authors (V.R. and T.V.-V.). The data are not yet publicly available due to temporary institutional embargo.
